# AI Applications in Adult Stroke Recovery and Rehabilitation: A Scoping Review Using AI

**DOI:** 10.3390/s24206585

**Published:** 2024-10-12

**Authors:** Isuru Senadheera, Prasad Hettiarachchi, Brendon Haslam, Rashmika Nawaratne, Jacinta Sheehan, Kylee J. Lockwood, Damminda Alahakoon, Leeanne M. Carey

**Affiliations:** 1 Centre for Data Analytics and Cognition, La Trobe Business School, La Trobe University, Melbourne, VIC 3086, Australia; i.senadheera@latrobe.edu.au (I.S.); p.hettiarachchige@latrobe.edu.au (P.H.); b.nawaratne@latrobe.edu.au (R.N.); d.alahakoon@latrobe.edu.au (D.A.); 2 Occupational Therapy, School of Allied Health, Human Services and Sport, La Trobe University, Melbourne, VIC 3086, Australia; b.haslam@latrobe.edu.au (B.H.); j.sheehan@latrobe.edu.au (J.S.); k.lockwood@latrobe.edu.au (K.J.L.); 3 Neurorehabilitation and Recovery, The Florey, Melbourne, VIC 3086, Australia

**Keywords:** artificial intelligence, neurorehabilitation, stroke rehabilitation, recovery, therapy, machine learning

## Abstract

Stroke is a leading cause of long-term disability worldwide. With the advancements in sensor technologies and data availability, artificial intelligence (AI) holds the promise of improving the amount, quality and efficiency of care and enhancing the precision of stroke rehabilitation. We aimed to identify and characterize the existing research on AI applications in stroke recovery and rehabilitation of adults, including categories of application and progression of technologies over time. Data were collected from peer-reviewed articles across various electronic databases up to January 2024. Insights were extracted using AI-enhanced multi-method, data-driven techniques, including clustering of themes and topics. This scoping review summarizes outcomes from 704 studies. Four common themes (impairment, assisted intervention, prediction and imaging, and neuroscience) were identified, in which time-linked patterns emerged. The impairment theme revealed a focus on motor function, gait and mobility, while the assisted intervention theme included applications of robotic and brain–computer interface (BCI) techniques. AI applications progressed over time, starting from conceptualization and then expanding to a broader range of techniques in supervised learning, artificial neural networks (ANN), natural language processing (NLP) and more. Applications focused on upper limb rehabilitation were reviewed in more detail, with machine learning (ML), deep learning techniques and sensors such as inertial measurement units (IMU) used for upper limb and functional movement analysis. AI applications have potential to facilitate tailored therapeutic delivery, thereby contributing to the optimization of rehabilitation outcomes and promoting sustained recovery from rehabilitation to real-world settings.

## 1. Introduction

Stroke remains a significant cause of long-term disability worldwide. Every year, 16 million people are affected by stroke globally, with approximately five million of them left with a permanent disability [1]. Stroke often leads to significant deficits in physical and cognitive functions, thus constituting a considerable burden on healthcare systems and society in terms of healthcare expenses, loss of independence, and decreased quality of life. A large proportion of community-living stroke survivors live with chronic and complex health needs which are often unmet [2]. This can result in significant impacts on health and productivity, in addition to economic and societal costs, with the largest cost component being productivity costs and the biggest impact being loss of healthy life and burden of disease [3].

The effects of stroke, including physical, cognitive and emotional sequelae, are complex and can be life-changing, which calls for comprehensive rehabilitation strategies [3]. Among the different impairments, sensorimotor, cognitive, speech and functional impairments are particularly challenging and can manifest in various forms, such as hemiplegia, somatosensory disorders, aphasia, memory loss, loss of controlled use of the upper limbs and reduced independence and quality of life [4,5].

Stroke rehabilitation, which relies on neuroplasticity to restore brain functions, is a comprehensive and multidisciplinary approach to helping stroke survivors regain lost functions, relearn skills and adapt to new limitations [6]. The process addresses the physical, cognitive and emotional challenges that arise from a stroke. Rehabilitation can occur from the acute phase immediately after a stroke and continue for months or even years [7]. Physical, occupational, cognitive and language therapies are commonly used in stroke rehabilitation. The intensity and focus of rehabilitation may vary depending on the individual’s needs and progress. The rehabilitation can be in two forms: inpatient rehabilitation at a hospital or rehabilitation facility or rehabilitation at the stroke survivors’ home. Of these, inpatient rehabilitation is reported to provide better outcomes than outpatient rehabilitation, possibly due to limited supervision by therapists in free-living conditions [8,9].

In contrast, stroke recovery is a lifelong process that involves ongoing recovery, overall adjustment and adaptation to life after a stroke. Recovery extends beyond the formal rehabilitation phase and encompasses ongoing changes in physical, psychological and social aspects [10]. The goals of recovery include adapting to any permanent changes resulting from the stroke, such as residual deficits or disabilities, emotional well-being, return to meaningful life activities and social reintegration. The initial stages of recovery may involve more intense efforts to improve and adapt to the immediate aftermath of the stroke, while later stages often focus on maintaining and optimizing the achieved gains in rehabilitation and focusing on quality of life.

In recent years, the use of artificial intelligence (AI) has become increasingly popular in healthcare settings, transforming how patients are assessed, diagnosed, monitored and treated [11]. Artificial intelligence is a technology that enables machines, such as computers and robots, to exhibit human-like intelligence, including the ability to solve problems and make decisions based on a set of rules, logical conditions or past experience [4]. The use of AI shows great promise for revolutionizing stroke rehabilitation through high-precision assessments, personalizing treatment plans, monitoring impairment, assisted rehabilitation and predictive delivery of healthcare in inpatient and home environments [12]. These advantages are mainly due to the high precision of AI to predict long-term recovery paths, assist in better therapeutic decision making as well as assist in the classification of neurological and functional impairments [12]. Artificial intelligence technologies like machine learning (ML), deep learning, computer vision and natural language processing (NLP) can systematize and accelerate the processing of vast and heterogeneous data derived from stroke patients, including clinical records, neuroimaging data, kinematic evaluations from sensor and robotic devices and brain and bioelectric signals [13]. Additionally, AI techniques can identify patterns humans cannot perceive, providing new insights into patients’ progress and individual needs [14], thus enabling tailored intervention.

Despite the potential benefits of AI technologies in enhancing post-stroke rehabilitation outcomes, various challenges persist, including issues related to skilled resources, equipment, costs, technology limitations, real-world clinical implementations and factors associated with its use, including ethical considerations. For example, delivery using AI-driven automated systems may be more expensive than traditional approaches, as skilled therapists are still involved in both automated and traditional approaches, while automated systems also need specialized equipment and analytical skills [9]. Furthermore, many AI-driven rehabilitation tools often suffer from issues related to poor explainability due to the technical “black box” nature of machine learning models [15].

In the literature, there is a broad exploration of how AI technologies are applied to stroke rehabilitation and recovery. Rahman and colleagues investigated AI-powered automated assessments and rehabilitation systems with a specific focus on robot-assisted and virtual reality (VR) [9]. Ma et al. [16] summarized the literature on AI applications in four main areas of stroke care: diagnosis, outcome prediction, treatment and rehabilitation. Another review on stroke care identified a decade of literature from 2012 to 2022 covering any aspect of stroke care, from diagnosis to rehabilitation and other stroke-related topics [13]. Applications of ML in multiple stroke-related problems, including prevention/risk factor identification, diagnosis, treatment and prognostication, were summarized by Sirsat et al. [1]. Furthermore, Choo et al. [17] discussed common ML techniques used in stroke rehabilitation. While several other reviews have already been conducted on the use of AI in stroke rehabilitation, with a focus on specific areas such as evaluation [4], monitoring and assistance [18], long-term outcome prediction [19,20], assessment [21], robot assistance [22,23], gait [24], motor function [25], brain–computer interfaces (BCI) [26], cognitive rehabilitation and speech [27,28], the existing literature lacks an overall review on the progression of AI-assisted adult stroke rehabilitation and recovery over time, in particular with regard to the upper limbs and their functional use. Therefore, the aim of the current review is to explore the evolution of AI applications in stroke rehabilitation and recovery in adults over time, delineate existing clinical usage of this evidence and highlight areas for future clinical development with a special focus on AI-assisted rehabilitation of the upper limb and its functional use.

## 2. Materials and Methods

This scoping review followed the process outlined in the Preferred Reporting Items for Systematic reviews and Meta-Analyses extension for Scoping Reviews (PRISMA-ScR) statement [29].

### 2.1. Eligibility and Exclusion Criteria

Peer-reviewed articles published in the English language, including journal articles, conference papers, clinical trials, case reports, reviews and book chapters, reporting on the use of AI-based applications for stroke rehabilitation and recovery of adult stroke survivors (age > 18 years) with any type of stroke (ischemic or hemorrhagic) were included. Branches of AI (e.g., machine learning, deep learning, reinforcement learning) with virtual and physical applications were included. Applications such as VR, robotics, sensors and mobile apps were included if they involved AI techniques. Studies that included AI encompassed impairment assessment, intervention, monitoring, outcome prediction, telerehabilitation, personalized treatment and long-term recovery. Studies involving non-human subjects and those reported in languages other than English were excluded. We also excluded all types of protocols, conference abstracts, dissertations, editorial letters, guidelines, websites and other references that were not peer-reviewed.

### 2.2. Information Sources and Search Strategy

Potential articles were identified by conducting a comprehensive search on MEDLINE, CINAHL, EMBASE, CENTRAL, PEDRO Web of Science, IEEE Xplore, ACM Digital Library, SCOPUS, SpringerLink and Wiley Online Library. We developed search keywords based on Medical Subject Headings (MeSH) terms. The search was then expanded and adapted to other databases based on a domain vocabulary developed (I.S. and P.H.) and reviewed by B.H., R.N., D.A., and L.M.C. (Table 1). The vocabulary included both clinical and computational terms (Appendix A). The search was carried out on the full texts to gather articles up to early 2024. This process was conducted on 22 January 2024.

### 2.3. Data Extraction and Screening

Data collected from academic databases included characteristics such as title, abstract, year of publication, index terms and keywords as identified in the article by the authors. These data were selected for their availability and based on the expectation that key topic words should be captured in these sources. Duplicate records were then eliminated.

We used a multi-step, multi-method (programmatic and manual) approach to screen the search results. Firstly, a keyword search was conducted on the title, abstract, index terms and author keywords within the collected data to further filter out articles. Secondly, we employed a novel automated workflow using NLP techniques to assist manual screening. NLP is known as the application of computational techniques to analyze natural language, which includes textual data [30]. The automated screening was conducted by I.S., and the results were manually screened by further authors B.H., K.J.L. and J.S. to confirm inclusion and exclusion. Disputes were resolved by B.H. and L.M.C. The process is described in Supplementary Materials S1.

### 2.4. Data Analysis

We developed a clinical text-processing engine with an array of NLP-based algorithms for key term extraction and topic modeling and to create a topic ontology that was used to derive insights on how research evolved over time. For example, key term extraction is used to automatically identify and extract the most relevant and significant terms or phrases from a corpus of text to provide insights into its content and facilitate further analysis. The attributes used for the text analysis were the title, abstract, keywords and index terms.

#### 2.4.1. Topic Modeling for Identifying Research Themes

We used a topic modeling approach to first identify common clusters of research topics to reveal interpretable themes in the included articles. We employed the BERTopic technique to automatically cluster topics by grouping articles (title and abstracts) based on the semantic similarity of the content they described [31]. Result topics were then grouped into four research themes based on expert consensus regarding the topic clusters identified.

#### 2.4.2. Mapping into a Clinical Ontology

Further automatic analysis of the title and abstract was conducted using a pre-trained NLP model specialized in clinical text processing [32]. Rehabilitation-related information was extracted and mapped into MeSH ontology, and the results were then stored in a database for further processing and visualization using an interactive dashboard.

#### 2.4.3. Development of Interactive Dashboard

**Figure 1:**
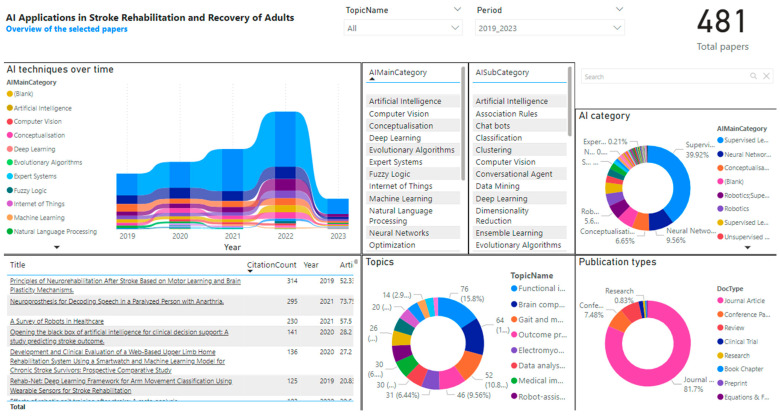
Screen captures of the interactive dashboard. Key data features depicted in the dashboard include: AI technology, clinical methodology, time-linked patterns and network analysis.

In order to conduct a detailed analysis of the available data, a sophisticated technique was devised using the Microsoft Power-BI (version 2.136) interactive dashboard. It included key terms, research themes, clinical ontology, AI and clinical categories, article types and journal rankings. Through the use of interactive visualizations, the dashboard facilitated the exploration of data insights, thereby highlighting important associations, insights and patterns. Moreover, the developed dashboard was utilized for analysis of research over time, allowing for a comprehensive evaluation of the evolution of different AI techniques and the progression of research objectives in adult stroke rehabilitation and recovery. Figure 1 showcases a screen capture of the dashboard that was developed. The dashboard is available for readers to access online at https://bit.ly/3X4k5HR, accessed on 20 August 2024.

### 2.5. Evaluation of AI for Upper Limb Rehabilitation

As an exemplar of how the data may be used, and based on the interest of the authorship team, we selected the top ten notable articles that focused on AI technologies in upper limb rehabilitation for data extraction, full-text review and evaluation. Studies that demonstrated clear applicability in clinical settings and presented validated outcomes with high article impact were considered for the selection. These articles are interpreted in the discussion.

## 3. Results

### 3.1. Search Results and Selection

**Figure 2:**
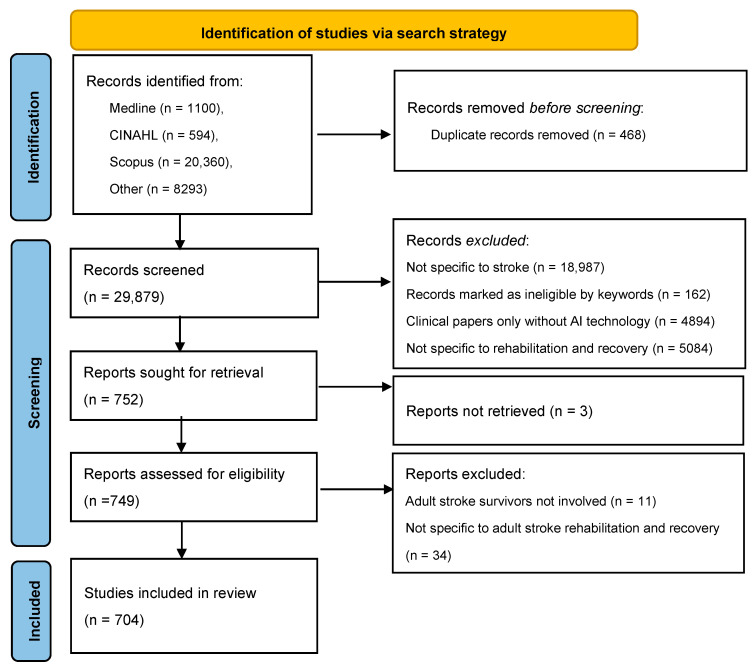
Flow diagram of studies included in the scoping review.

Figure 2 illustrates the search results and inclusion. The number of articles retrieved by the search process was 30,347, with 1100 articles from PubMed, 594 from CINAHL, 1834 from ProQuest, 521 from Web of Science, 2384 from Embase, 2341 from ACM, 61 from IEEE Xplore, 1080 from SpringerLink, 72 from Wiley Online and 20,360 from Science Direct. Once duplicate records (n = 468) were removed, 29,879 articles were screened. During the screening of titles, abstract keywords and index words, a total of 162 articles were excluded as they were not related to human studies. A total of 18,987 articles were then excluded as they did not match keywords from the developed vocabulary of AI, stroke rehabilitation and recovery. The multi-method screening resulted as follows: 5084 articles were excluded as they did not relate to stroke rehabilitation or recovery, and 4894 articles were excluded as they did not use AI technologies. This resulted in 752 articles for retrieval. Three articles were excluded as full texts were not available, thus 749 articles were assessed for eligibility by full text. Studies involving no adult participants (n = 11) and not specific to adult stroke rehabilitation and recovery (n = 34) were excluded. A total of 704 articles were included in the review.

A total of 310 (44%) articles were related to upper limb rehabilitation and recovery across themes. Ten notable articles were selected for full-text review, with a special focus on upper limb rehabilitation and its functional use. The selected studies reflected a cross-section of identified themes.

### 3.2. Overview of Results

It is noted that 564 (80%) were journal articles, 77 (11%) were conference papers, 44 (6%) articles were reviews, 2 (<1%) were book chapters, 2 (<1%) were equations and formulas, 11 (2%) were experimental studies and 16 (2%) were randomized controlled trials. Furthermore, 367 (52%) articles were published in Q1 journals, 165 (23%) articles in Q2 and 69 (10%) articles in Q3 journals.

We present the findings of this review from multiple perspectives, including research themes, clinical applications and utility, commonly used AI techniques and progression of AI technology applications over time.

### 3.3. Research Themes on AI Technology Approaches in Adult Stroke Rehabilitation and Recovery

**Figure 3:**
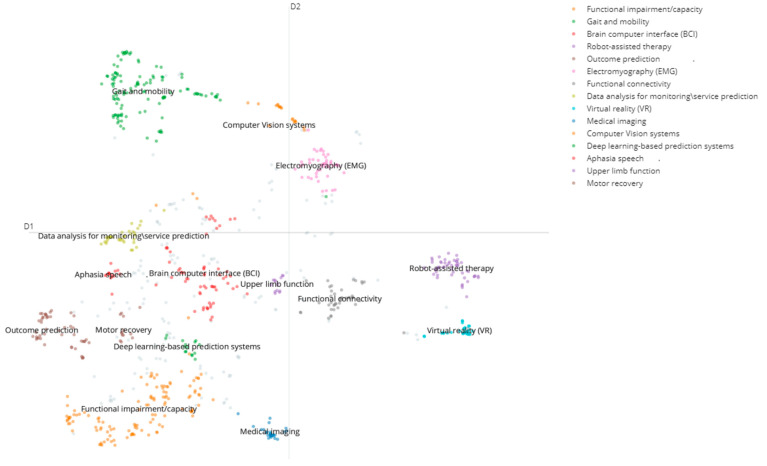
Topic clusters to characterize the research landscape of AI applications in adult stroke rehabilitation and recovery. Clusters are represented by different colors.

Adult stroke rehabilitation has a growing interest in the integration of AI. Our topic clustering analysis identified fifteen topic clusters that helped to characterize studies and four major research themes in the field of AI applications in adult stroke rehabilitation and recovery (Figure 3; Table 2). The identified major themes included: impairment, assisted intervention, prediction and imaging and neuroscience. Table 2 shows the identified research topics under each major theme, with an indication of the number of articles.

#### 3.3.1. AI-Based Applications in Post-Stroke Impairments

The impairment theme comprised common research topics such as functional impairment and capacity, gait and mobility, electromyography (EMG) for assessing motor impairment, upper limb function, and speech impairments. Of these, functional impairment and capacity (16%) and gait and mobility (12%) were the most discussed topics.

Studies categorized under functional impairment and capacity (n = 115) were directed toward impairment assessment; clinical score emulation [9,33]; assisted intervention [34] and predicting function, including activity and participation [4,35,36], focusing primarily on motor function. Artificial intelligence techniques like supervised learning-based classification and artificial neural networks (ANN) have been used for functional assessment, disability prediction and recovery, including motor recovery evaluation [37], balance control [38], task-oriented exercise quality [39], swallowing [40] and postural behavior analysis [41]. Furthermore, motor function recovery research included functional motion quality [42], compensation assessment [43] and movement coordination [44]. Moreover, studies focused on cognitive impairment explored the remote assessment of cognitive impairment [45] and computer-assisted adaptive cognitive rehabilitation [46]. In these studies, various data from sensor devices such as functional magnetic resonance imaging (fMRI), inertial measurement units (IMU), accelerometers, pressure sensors and myography sensors, as well as clinical data such as the Fugl-Meyer Assessment (FMA) scale, Mini-Mental State Examination (MMSE) and Montreal Cognitive Assessment (MoCA) were used to assess and predict rehabilitation outcomes and the level of change in functional impairment. AI algorithms have been applied in novel virtual rehabilitation systems that automatically recognize psychological states to promote therapy compliance [47]. Such applications assist in providing feedback on movement performance during exercises and objectively measuring physiological signals, thus offering new methods for monitoring functional capacity changes in stroke survivors.

Of the included articles, 12% (n = 83) discussed the topic of gait and mobility, with the goal of analyzing gait patterns and mobility using wearable sensor devices and motion capture systems in assessing walking and balance, assisted gait training and rehabilitation, monitoring and predicting the outcomes [48,49,50] regarding stroke survivors’ independence and functional mobility, thereby assisting therapists in treatment planning and reducing decision-making time. Supervised learning and ANNs have been employed to detect biomechanical gait abnormalities like hemiparetic gait [51], foot drop [52] and ankle joint stiffness [53]; to detect gait phases like swing [52] and to quantify lower limb motor dysfunction [54]. The integration of AI with wearable and motion sensing sensor technologies has allowed for a holistic approach to gait and mobility monitoring and assessment in stroke survivors, covering aspects such as spatiotemporal gait characteristics, muscle function and balance control. Various wearable sensors like IMU, accelerometers and pressure sensors [55,56,57] were employed in the studies, aiding clinicians in diagnosing and tailoring rehabilitation strategies. Kaczmarczyk et al. [58] used ANNs to classify participants into pathological gait groups using computed tomography (CT) scan parameters. Koenig et al. [59] used the linear discriminant analysis (LDA) technique to quantify the cognitive load by using psychophysiological measurements and gait performance, adapting virtual ambulatory task difficulty levels in real-time during gait training. These assessments aid in understanding the impact of stroke on gait and mobility, assisting in personalized rehabilitation strategies. AI-based fall assessment systems for older adults have shown significant improvements in gait speed and mobility, highlighting the potential for early-stage interventions [55]. Another study used human activity recognition technologies to enable continuous monitoring of ambulatory function and participation in daily activities [49,60]. By accurately categorizing gait conditions, interventions can be tailored to meet individual needs for improved recovery, enhancing the effectiveness of gait rehabilitation programs in outpatient settings or home environments.

Upper limb impairment with sensor technologies was a specific focus of 4% (n = 28) of the articles. Studies categorized into upper limb function covered a range of topics, including functional assessment [61,62], rehabilitation and outcome prediction [63,64,65]. AI algorithms and sensors like IMUs and depth-sensing cameras like Kinect have been used for clinical score emulation, such as the FMA, providing efficient and objective measures of upper limb function [61,62]. Furthermore, supervised learning-based classifiers such as regression models have been used for identifying functional and non-functional motions to quantify real-life upper limb use with high accuracy, aiding in motor recovery assessments and treatment planning [66]. Multi-sensor-based rehabilitation training, including home-based telerehabilitation, utilizes low-cost sensors such as Kinect and multimodal AI for repetitive training and evaluation, promoting accessibility and adherence to therapy [62]. Wearable sensors, including IMU and accelerometers, have been used to objectively identify task types [63,67] and movement types and quality [61,67], achieving high accuracy in task identification and movement quality classification. These sensors enable continuous monitoring of upper limb movement quality and impairment levels, facilitating personalized interventions and progress tracking. Furthermore, Friedman et al. [68] developed a customized wearable device manumeter for monitoring daily use of the wrist and fingers. Combining sensor data and machine learning, smartphone-based systems have also been experimented to facilitate independent and remote rehabilitation, reducing costs and improving compliance [64].

Forty-four articles (6%) focused on electromyography, often in the context of assessing and monitoring motor impairment. EMG signals provide valuable information about muscle activity and voluntary movement, making them suitable for controlling rehabilitation and assistive devices, including robotic applications. In the assessment context, supervised learning techniques with surface EMG and wearable sensors like IMUs have been used in evaluating post-stroke spasticity levels [69], indicating the utility of AI techniques for objective spasticity quantification. Additionally, in the assessment of motor deficits, multimodal AI techniques applied to kinematic and surface EMG data help to quantify abnormal synergies in stroke-affected upper limbs, demonstrating the use of supervised learning methods in assessing motor impairments [70]. Furthermore, the novel application of EMG for detecting motor imagery of swallowing and tongue protrusion offers a promising solution for post-stroke dysphagia rehabilitation [71]. In the context of rehabilitation, AI-integrated EMG-driven systems such as robot hands have been developed to aid upper extremity rehabilitation in chronic stroke patients [72], demonstrating improvements in motor function. Yang et al. [73] developed a ML-powered wearable smart robotic arm to discriminate forearm gestures for assisted rehabilitation training. For example, Cai et al. [74] used supervised learning techniques with surface EMG interfaces to classify muscular activities in real-time, achieving high accuracy in recognizing hand movements with an assisted self-rehabilitation device. Another wearable assistive device controlled by surface EMG signals was designed to aid in post-stroke hand function recovery, showcasing the use of ANNs for EMG-to-muscle activation modeling and EMG-based control strategies for finger exoskeletons [75]. EMG signals are also used in lower limb function assessment [76] and gait monitoring [77], with AI classifiers providing high accuracy in identifying different activities and myoelectric biomarkers, which is beneficial for targeted muscular rehabilitation.

We found that 3% (n = 23) of articles reported speech impairment. NLP techniques are heavily used in aphasia and dysarthria assessment and speech therapy to analyze linguistic patterns, speech production and comprehension abilities in order to create personalized speech therapy exercises [78,79,80,81]. Moses et al. [81] developed a speech recognition system using NLP and ANNs with BCI to make predictions from individuals’ neural activity in real-time for participants with anarthria. Furthermore, ANNs have been used to evaluate tone production in Mandarin-speaking patients with post-stroke dysarthria, demonstrating comparable recognition rates compared to human listeners [82]. Moreover, Ye et al. [83] explored pathological voice recognition for dysarthria assessment using ANN techniques like convolutional neural networks (CNNs) and long short-term memory neural networks (LSTMs). Another study used ML classifiers with structural MRI to predict aphasia type [84]. These advancements not only streamline the assessment of speech quality, but also address the dynamic nature of speech evaluation techniques.

#### 3.3.2. AI-Based Systems in Assisted Intervention

Under the theme of assisted intervention, common topics included BCI, robot-assisted therapy, virtual reality (VR), computer vision and deep learning-based systems.

A large proportion, 14% (n = 100), explored the use of BCI to establish a communication channel between the brain and computers, enabling stroke survivors to modulate their sensorimotor rhythms purposefully, providing sensory feedback on ongoing brain oscillation [85]. BCI allows participants to control computers, prosthetic limbs, or other assistive devices using neural signals in AI-driven motor, cognitive and sensory assessment, as well as in rehabilitation and outcome prediction [85,86,87,88,89,90,91]. In a rehabilitation context, AI-driven BCI applications utilized electroencephalogram (EEG) data to decode motor intentions and facilitate neurofeedback training, which has been shown to improve motor function in stroke survivors [87]. Recent advancements have also focused on combining BCI and EEG technologies with functional electrical stimulation (FES) to enhance upper limb motor recovery, showing greater efficacy compared to conventional FES application in chronic phase [92]. For example, Abdallah et al. [93] adapted a fuzzy logic-based pain detection approach with FES and EMG signals for enhancing the efficiency of upper limb exoskeleton in rehabilitation. Furthermore, AI-driven BCI and EEG can achieve high recognition rates in differentiating specific movement disorders, such as shoulder versus elbow torque [94] or finger-tapping [88] for neurofeedback therapy. Yanagisawa et al. [95] used BCI with electrocorticography to control a prosthetic arm in real-time and classified arm movements using the support vector machine (SVM) classification algorithm for restoring upper limb motor function. The integration of AI and robotic coaches in physical rehabilitation therapy has been explored to provide systematic management and to improve self-efficacy and motivation during rehabilitation sessions [96]. Moreover, BCI with AI-enabled VR games has been employed for rehabilitation purposes [92], allowing for the identification of specific motor imagery being performed by stroke survivors. ANNs with BCI and EEG were used in motor imagery outcome prediction and prognosis [89]. One study utilized k-nearest neighbors (KNN) and probabilistic neural network AI algorithms with EEG signals to predict emotional states in stroke patients [97], showcasing a correlation between brain damage location and impaired emotion recognition.

AI applications in robot-assisted motor rehabilitation therapies were discussed in 9% (n = 61) of the articles. These AI-driven therapeutic robots have significantly advanced the field by enabling automated assessment, intelligent assistance and outcome prediction in the rehabilitation process [98,99,100,101]. We noted investigations into the use of robotic devices equipped with AI for sensorimotor and upper-limb recovery after stroke. These robotic devices included exoskeletons and assistive or demonstrative robots that can assist patients in regaining control over the affected limbs, providing repetitive and task-specific training to improve motor function of upper and lower limbs [102,103]. In the context of motor rehabilitation, studies have explored the effects of robot-assisted gait training in combination with physiotherapy [101], showcasing high accuracies in outcome prediction in gait recovery and functional ambulation. Studies have explored using functional near-infrared spectroscopy signals with supervised learning to detect brain activity during lower limb motor preparation in chronic stroke patients, showing promise for BCI applications in robot-assisted rehabilitation [104]. In the context of assessment, robotics devices and wearable sensors have been used to quantify upper limb motor control using regression analysis techniques [99]. These applications aid in establishing personalized rehabilitation goals and resource allocation based on prognostic factors and quantitative data from robot-assisted training sessions.

Virtual reality technology has experienced significant advancements and widespread adoption over the past decade. Five percent (n = 33) of studies have explored the integration of VR technologies with AI to create immersive and engaging rehabilitation environments. These virtual environments can be used for motor assessment, therapeutic exercises, balance training, motor recovery and upper limb function rehabilitation, thereby enhancing patient independence and quality of life [105,106,107,108]. Virtual reality applications such as three-dimensional serious virtual games have been deployed in home-based settings to encourage full-body and upper limb exercises while using ANN techniques to quantify the performance [106]. One study used integrated depth sensors with a VR-based rehabilitation system and evaluated the quality of the physiotherapy exercises using ANNs [107], showing the feasibility of a remote physical training monitoring environment for upper limb rehabilitation. Furthermore, potential cognitive rehabilitation through VR, as seen in the VIRTUE application [108], has shown benefits in problem solving, memory, and task sequencing, with gamification techniques enhancing patient motivation and reducing hospital stay durations. EMG-integrated VR-based training systems employing ANNs have demonstrated the potential for adaptive hand movement training engagement [109]. Furthermore, supervised learning techniques have been used to predict outcomes of VR-based therapy techniques such as exergames [110], enabling physiotherapists to monitor and predict their patients’ progress.

Computer vision systems were investigated in 23 studies (3%). Computer vision-based assessment and assisted intervention have emerged recently, offering advanced techniques to enhance rehabilitation outcomes. This technology enables the markerless, continuous monitoring and assessment of individual performance through non-invasive methods, such as the use of video cameras, depth cameras, and motion capture systems [111,112,113]. Moreover, these systems can provide real-time objective assessment and feedback on motor function, promoting more effective and engaging telerehabilitation [111]. Supervised learning algorithms like SVM have been used to detect upper limb compensatory movements during computer vision-based rehabilitation therapy [111]. Sucar et al. [114] developed a computer vision-based rehabilitation system for practicing repetitive arm movement exercises at home or at the clinic. This system used a pressure sensor for hand and finger rehabilitation and detected trunk compensation by tracking the head of the participant. Han et al. [115] developed a novel upper limb rehabilitation system for monitoring hand motion and assessed grip using a custom marker device. In the assessment context, studies have explored computer vison-based automated approaches employing video and depth cameras to monitor movements during tasks like the box and blocks test [112]. Computer vision is also applied in lower limb rehabilitation by integrating depth cameras and rehabilitation robots [116] to measure the range of motion of lower limb joints, improving objectivity and efficiency in the rehabilitation process.

Three percent of the studies (n = 21) focused on deep learning-based approaches in post-stroke motor function assessment and rehabilitation. Panwar et al. [117] developed an ANN-driven framework called “Rehab-Net” to classify upper limb movements of stroke survivors with high accuracy, providing a measure of rehabilitation progress. This framework utilized wrist-worn accelerometer sensors and employed low-complex CNN models for monitoring. Furthermore, deep learning techniques have been applied in computer vision-based monitoring systems. One study [118] proposed a computer vision-driven automated motor function rehabilitation system that guides stroke survivors with real-time feedback in physiotherapy exercises at home. This study integrated a Microsoft Kinect version 2 depth camera with hybrid deep learning approaches, experimenting with multiple ANNs such as CNN, LSTM, recurrent neural networks (RNNs) and gated recurrent units (GRUs). Another study [119] proposed a computer vision-based markerless pose estimation for gait analysis by tracking five key body points. A CNN was trained to estimate clinically relevant gait parameters, such as cadence and walking speed, from video data, and this approach enabled quantification of gait metrics even in uncontrolled environments. Deep learning techniques were used in lower extremity rehabilitation. For example, Rose et al. [120] employed a deep reinforcement learning technique for adaptive control of the lower body exoskeleton during gait, improving participation and motor learning. Moreover, innovative approaches like the body weight support locomotion training platform with ANN-driven gesture recognition demonstrated high accuracy in controlling mobile support platforms for lower limb rehabilitation [121].

#### 3.3.3. AI-Based Systems in Outcome Prediction and Prognosis

AI-based prediction research in adult stroke rehabilitation and recovery covers several automated systems, including neurological outcome prediction, functional motor recovery prediction and clinical data analysis for monitoring and service prediction. The prognosis is the clinician’s assessment of how a stroke survivor will recover by forecasting the level of change in assessment scores. In contrast, outcome is the end result of an intervention and a consequence of intervention decisions made by the therapist. With the increasing availability of multifactorial data from diverse sources, supervised learning predictive algorithms, such as SVM and random forests, have shown high accuracy over classical methods in recovery prediction and prognosis [122]. Such applications allow data-driven evaluation based on biomechanical, physiological, biochemical or metabolic biomarkers in characterizing motor, cognitive and sensory recovery trajectories and making informed decisions about treatment approaches and long-term recovery planning [123].

Outcome prediction refers to the use of data to forecast the effectiveness of post-stroke interventions, such as the level of change in functional abilities, quality of life and long-term health status, which aids in long-term care planning and setting realistic expectations. Seven percent (n = 50) of studies focused on outcome prediction, particularly on acute ischemic stroke survivors, across different areas such as functional independence, motor abilities, cognition, depression and mortality [15,124,125,126], utilizing AI techniques like ANNs, SVM, random forests, regression and Bayesian networks. ANNs have been employed to predict discharge Functional Independence Measure (FIM) scores for stroke survivors with moderate disability [127], aiding in discharge planning in rehabilitation programs. Bayesian network classifiers have been used for predicting functional independence and post-stroke mortality, showing high accuracy in outcome prediction [128]. In addition, supervised learning techniques, such as SVM, random forest and logistic regression, have been utilized to predict the long-term functional outcomes of acute ischemic patients undergoing endovascular treatment for large vessel occlusion [129]. Monteiro et al. [130] employed algorithms such as SVM, decision tree, random forest and Xgboost in accurately predicting the functional outcomes of ischemic stroke survivors, aiding in treatment decisions. Moreover, another study investigated explainable AI methods for traditional ML algorithms and ANNs with stroke biomarkers in predicting recovery outcomes in the acute ischemic stage at 90 days, showing high accuracy in predicting modified Rankin scale (mRS) scores [15]. Patel et al. [124] employed the random forest technique with wearable accelerometer data recorded while a stroke survivor performed the motor tasks pertaining to the Functional Ability Scale to predict motor rehabilitation outcomes. Furthermore, SVM and ANNs have been applied in prognostic modeling of endovascular intervention in acute ischemic stroke to predict outcomes, demonstrating AI’s role in guiding treatment decisions [131]. These AI-driven predictive models have the potential to guide personalized rehabilitation interventions tailored to individual patient needs, thereby optimizing post-stroke recovery strategies.

AI applications for identifying key prognostic factors leading to outcomes through data analysis for monitoring and service prediction were found in 6% (n = 39) of studies. Service prediction in post-stroke rehabilitation refers to forecasting the types, intensities, and duration requirements of rehabilitation services that a stroke survivor will need to optimize their recovery and quality of life. Accurate prediction of clinical impairment following therapy is key in prescribing appropriate therapeutic strategies [132]. This aids in planning the transition from the rehabilitation facility to community-based care by determining the level of ongoing support required and allocating resources efficiently, ensuring that patients receive appropriate care at the right time. ML techniques such as logistic regression, SVM, random forest and ANNs have been employed to predict outcomes based on various factors, including initial clinical assessments [132]. These models have shown promise in prognosticating activities of daily living (ADL), upper limb motor function, cognitive impairment and even long-term mortality [132,133,134], which are essential for therapeutic planning. For instance, predicting the Barthel Index scores [133] using ML algorithms has enabled healthcare providers to efficiently estimate a patient’s functional independence at discharge, upon initiating rehabilitation. Both ML and ANNs have been used to improve prediction, particularly in understanding influential factors, such as the rehabilitation requirement for an ankle–foot orthosis [135] and toileting independence [136]. Furthermore, explainable AI methods have improved transparency in AI predictions, helping clinicians understand the key predictive factors and leading to more informed decision making [137]. These AI applications in stroke rehabilitation support a multidimensional approach that considers motor, cognitive and neuropsychological predictors, thereby enabling a transition toward patient-centred care and precision rehabilitation [138,139].

Motor recovery prediction was investigated in 2% of studies (n = 14), specifically focusing on upper limb function in the chronic stage. One study predicted upper limb motor recovery after stroke employing SVM and using voxel-wise lesion likelihood values, demonstrating the capability to classify stroke survivors as good or poor recoverers [140]. This aids in planning treatments and stratification in restorative clinical trials. AI techniques like ANNs have achieved high accuracy, harnessing baseline assessments, including motor activity logs, tactile sensation tests and cognitive measures, to predict patient responses to therapies like constraint-induced movement therapy (CIMT) [141]. Furthermore, studies have used ML regression algorithms leveraging wearable accelerometer sensor data to more accurately model individual responses to rehabilitation interventions in real-world settings [142].

#### 3.3.4. AI-Based Systems in Imaging and Neuroscience

AI applications in medical imaging and neuroscience offer improvements in diagnostic and therapeutic processes. We observed two major topics: medical imaging and functional connectivity, which provide detailed insights into brain reorganization, enabling targeted interventions that improve motor, cognitive and sensory recovery.

Functional connectivity involves the reorganization of neural circuits and networks to facilitate the recovery of motor and cognitive functions in post-stroke rehabilitation. Six percent of the studies (n = 40) focused on functional connectivity. Assessing functional connectivity, particularly in the acute phase, has been an area of interest. Utilizing resting-state fMRI data and ML techniques like SVM and independent component analysis (ICA) has demonstrated the ability to classify motor impairments and assess predictive post-stroke recovery outcomes [143]. Vahdat et al. [144] demonstrated high accuracy in distinguishing patients with motor deficits and predicting therapeutic outcomes, underlying the significance of interhemispheric connectivity and sensorimotor network reconfiguration. When applied to cognitive and motor rehabilitation, AI facilitated the identification of crucial network patterns within brain structures, helping to tailor rehabilitation protocols to individual recovery potentials. For example, in upper limb recovery, the use of SVM to classify natural recovery using connectomes based on structural tractography has delineated networks relevant to motor improvement, enabling precision-based neurorehabilitation strategies [145]. Hoo et al. used an SVM classifier with EEG microstate-based functional connectivity analyses to aid in understanding motor function interventions [146]. In addition, Gaizo et al. presented an AI-driven analysis of language networks in aphasic patients that provided insights into the dynamic evolution of functional connectivity, enabling better prediction of aphasia severity [147]. One study has employed principal component analysis (PCA) analysis and SVM classifier with fMRI to identify discriminative functional changes of BCI therapy in enhancing motor recovery in stroke survivors with upper limb deficits [148]. By monitoring changes in resting-state functional connectivity pre and post therapy, such analysis not only provides evidence for motor rehabilitation efficacy, but also facilitates non-motor brain networks recovery, emphasizing the potential of BCI interventions in promoting neuroplasticity post-stroke.

Four percent of studies (n = 30) explored AI applications in medical imaging; they specifically employed supervised learning and ANN technologies. In the context of assessments, ANN algorithms, including feed-forward neural networks and CNNs, have shown efficacy in automating the segmentation and analysis of brain lesions on CT and MRI, including diffusion-weighted imaging, offering better accuracy and efficiency compared to traditional methods [149,150,151]. These models can predict the presence and extent of infarctions, improving the speed and reliability of diagnostic processes [150]. In the context of outcome prediction, ANN algorithms like CNNs, ResNet, LSTM and auto-encoder network models have been used to integrate clinical and imaging data to forecast motor, cognitive, and speech impairment and recovery, as well as functional outcomes using CT and MRI scans [151,152,153]. Moreover, combining imaging data with clinical metadata using multimodal ANNs has enabled precise prognostic predictions, as seen in studies forecasting the modified Rankin Scale (mRS) scores, contributing to improved acute and long-term stroke management strategies [154].

### 3.4. Commonly Used AI Techniques

**Figure 4:**
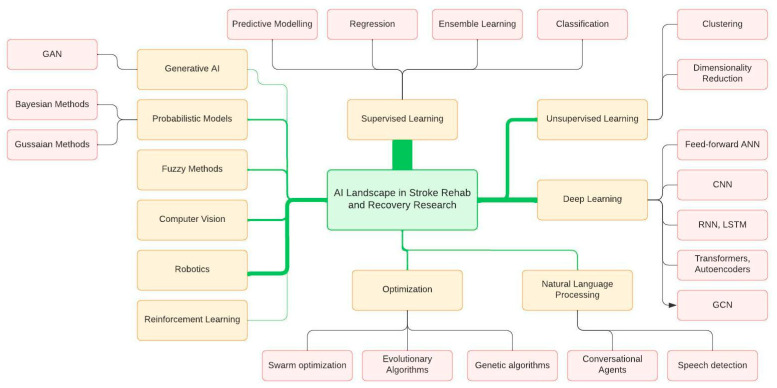
Landscape of AI techniques in stroke rehabilitation and recovery. The thickness of the lines indicates the usage of major categories of AI techniques. Yellow boxes indicate AI technique categories, and red boxes indicate specific AI techniques.

The AI landscape of commonly used AI techniques in stroke recovery and rehabilitation research can be mapped in several ways, each with its own techniques and applications (Figure 4). Our analysis indicated a significant research focus on AI applications in motor and upper limb rehabilitation, as well as gait and mobility and speech rehabilitation (Table 2). These applications include the use of major branches of AI technology, such as traditional ML, deep learning, NLP, reinforcement learning, computer vision, fuzzy logic, expert systems, optimization techniques, generative AI and robotics.

Traditional machine learning algorithms can be categorized under two major categories: supervised and unsupervised [17]. In supervised learning, the predicted outputs are known and used to train the models. The labeled training data helps in predicting outcomes via techniques such as classification, regression, predictive modeling and ensemble methods. In unsupervised learning, the desired output is unknown, and the objective is to discover structure in the data, not to generalize a mapping from inputs to outputs. This technique is used to discover hidden patterns in data without human intervention and relies on dimensionality reduction, clustering and association rule mining.

Deep learning consists of ANN, a mathematical and computational model that is inspired by the structure and functional aspects of biological neural systems [18]. ANNs consist of interconnected nodes (artificial neurons) processing information using a connectionist computational approach. This network adaptively changes its structure based on external or internal information, which flows during the learning phase, forming a robust dynamic model of the complex relationships between inputs and outputs or patterns in data. Unlike traditional ML methods that require handcrafted predictive factors (features) as input, deep learning methods learn these features adaptively from the data. Commonly used ANN models include feed-forward neural networks, CNNs, LSTMs and RNNs. Although it is beyond the scope of this review to detail AI algorithms, more information is provided in Supplementary Materials S2.

Supervised learning and deep learning are the most frequently employed techniques, with AI being specifically applied in impairment assessment, assisted rehabilitation and outcome prediction and prognosis [99,105,130]. Within supervised learning, classification techniques, including SVM, random forests, decision trees, KNN and ensemble methods, are widely used. SVM is most common due to its good generalization ability for sequential data and not-too-large datasets [17,155]. Tree-like algorithms, including decision tree and random forest, are commonly used due to their interpretability and the ability to rapidly learn [17,155]. KNN is popular since it is free from assumptions about the underlying data distribution [155]. Regression methods such as linear and logistic regression are also employed often [129,133]. Probabilistic models, though less common, include Bayesian networks and Gaussian models, primarily utilized for outcome prediction and assessment [128,156]. Deep learning techniques, including feed-forward neural networks, CNNs, RNNs and LSTMS, are often used for classification and predictive modeling studies [117,152]. Artificial neural networks are commonly used with complex data structures. Unlike traditional machine learning algorithms that need handcrafted features, ANNs can automatically learn latent data patterns. More recently, advanced ANN algorithms such as graph convolutional networks (GCN), autoencoders and transformers have emerged in a few studies [153,157,158]. These networks have been used to recognize complex patterns and features, helping in tasks such as medical imaging analysis, movement analysis, detection of abnormalities and even BCI and rehabilitation robotic exoskeleton control. Studies have shown that deep learning methods can outperform traditional handcrafted feature-based ML methods like SVM, KNN, random forest, etc. [9,117]. Further, multimodal learning has often been employed with ANN methods to integrate information from different sensor data modalities to achieve high accuracies in AI models [51,154]. Wearable IMUs, accelerometers, depth and motion capture sensors, and deep learning techniques have opened new avenues for automated stroke assessment and rehabilitation, offering effective ways to support patients in self-training and motivation during the recovery process [45,62,63]. Such systems have demonstrated high agreement with therapist evaluations, suggesting a promising future for tailored automated rehabilitation systems.

Unsupervised learning algorithms, such as clustering, like k-means and self-organizing maps (SOM), and dimensionality reduction algorithms, such as PCA and independent component analysis, are applied in many studies [67,73,117,159]. NLP is specifically used in speech recognition and conversational chatbots, including in aphasia research to aid speech and language rehabilitation, providing valuable insights into rehabilitation effectiveness and patient experiences [81,160].

Reinforcement learning, a branch of AI that leverages the principles of computational reward and punishment, is utilized in robot and VR assisted rehabilitation, with techniques such as ANNs and deep Q-networks [103,161,162]. Fuzzy methods such as fuzzy logic, fuzzy neighborhood preserving analysis and fuzzy neural networks are employed specifically in robot-assisted therapy, EEG and VR [90,109,163]. Optimization methods like genetic algorithms, evolutionary algorithms and swarm optimization are less frequently studied [96,164,165]. Furthermore, generative AI techniques like generative adversarial networks (GANs) have been applied with BCI and EMG systems, focusing on motor imagery [166].

### 3.5. Time-Based Analysis of AI Terminology and Evolution of Topics in Stroke Rehabilitation and Recovery

**Figure 5:**
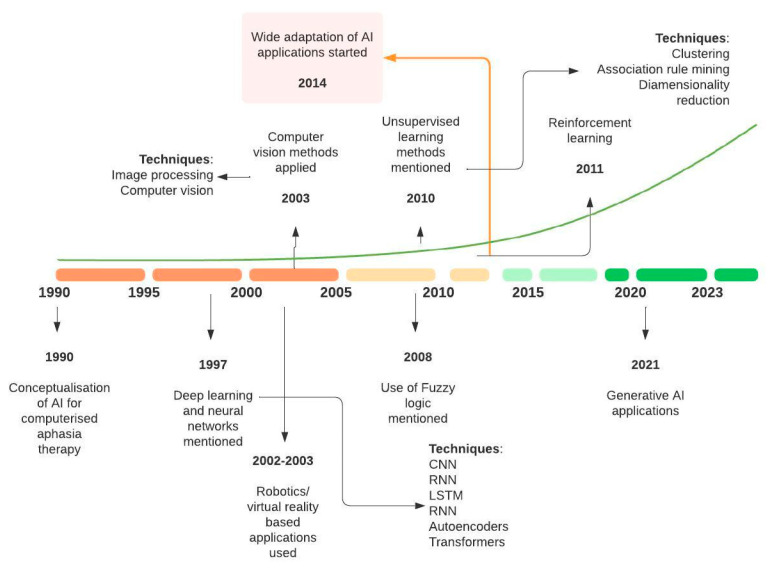
Progression of AI techniques applied in adult stroke rehabilitation and recovery research. Markers indicate the time of the first emergence of AI techniques.

We analyzed the emergence of AI technologies in stroke rehabilitation and recovery related studies. This indicated when certain techniques first appeared and how they evolved over time. The trend analysis indicated that the number of publications ranged from 1 in 1990 to 47 in 2023, with a significant increase after 2016, resulting in a peak (n = 162) during 2022. Based on the time-linked patterns identified by the first appearance and amount of research, we identified four time epochs of the research progression: (1) Conceptualization and Early Exploration (1990–2004); (2) Emergence of Machine Learning and Robotics (2005–2013); (3) Rise of Machine Learning and IoT (2014–2018); and (4) Current State: Personalized Rehabilitation and Telerehabilitation (2019–2024). Figure 5 shows the progression of research on AI applications in adult stroke rehabilitation and recovery.

**Figure 6:**
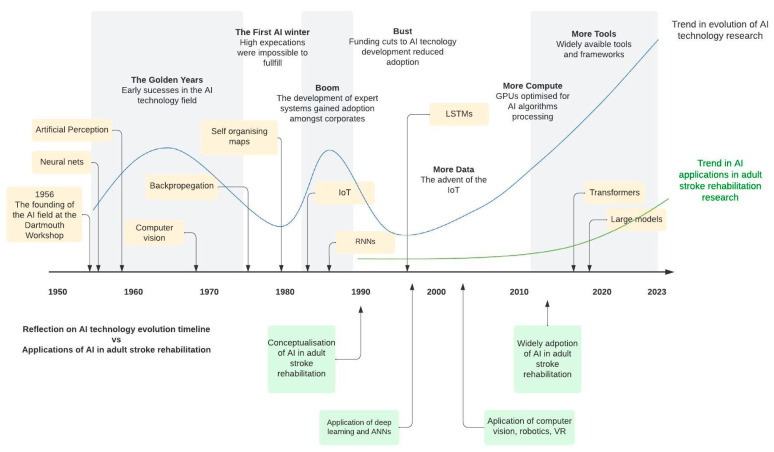
Timeline of AI applications in adult stroke rehabilitation compared to evolution of AI technologies. Yellow boxes (top) indicate the time of the invention of the AI technique. Green boxes (bottom) indicate the first application of AI technology in adult post-stroke rehabilitation and recovery.

Furthermore, we mapped the AI technology evolution timeline onto the above time epochs to understand the levels of technology infusion over time into adult stroke recovery research (Figure 6). In line with these time epochs, changes in the usage of previously existing techniques and the development of new research topics and AI techniques were observed.

During 1990–2004 (Conceptualization and Early Exploration), we observed early forays into supervised learning, deep learning and computer vision techniques applied in robot and computer-assisted rehabilitation, impairment assessment and outcome prediction. These programs often lacked sophistication, but they marked the beginning of integrating technology into rehabilitation settings.

The 2005–2013 period (Emergence of Machine Learning and Robotics) saw the growing applications of supervised learning, deep learning, computer vision and AI-driven rehabilitation robots, as well as the emergence of unsupervised learning, fuzzy logic and reinforcement learning techniques. Moreover, applications of wearable sensors emerged during this period. These technologies were applied in assisted rehabilitation and impairment assessment, specifically focusing on motor and upper limbs. Furthermore, several research topics emerged during this epoch, including gait and mobility, BCI, EMG, functional connectivity and data analysis for service prediction.

The time period of 2014–2018 (Rise of Machine Learning and IoT) marked a significant shift towards the fast-growing applications of supervised learning, deep learning and unsupervised learning, with growing applications of sensors, including wearables and robotics, into adult stroke rehabilitation and recovery. Of these, a major portion consisted of supervised learning and ANN applications in impairment assessment. Furthermore, these AI techniques, fueled by large amount of data, enabled the development of prediction and predictive care delivery. AI-driven rehabilitation robots and exoskeletons particularly focused on assisted therapies and stimulation. These models could identify patterns in patient data and predict optimal rehabilitation interventions and service delivery tailored to individual needs.

In recent years (2019–2023), we observed advanced AI applications in personalized rehabilitation and telerehabilitation experiences, enabling stroke survivors to engage in therapy sessions from their homes, enhancing accessibility to care and addressing geographical barriers. In particular, the COVID-19 pandemic revealed the need and opportunity for telehealth technologies in future stroke care delivery systems. During this period, the emergence of research topics like medical imaging, telemedicine and deep learning-based predictive systems were also observed. Notable emerging AI technologies identified include generative AI techniques, graph neural networks, transformers and autoencoders.

## 4. Discussion

To the best of our knowledge, there has been no prior exploration of the application of AI in stroke rehabilitation of adults that specifically highlights the evolution of clinical applications of AI technologies. Therefore, we conducted a scoping review of relevant studies to describe the evolution of and current research on AI technologies in stroke rehabilitation for adults. Our primary focus was on the use of AI for the evaluation of impairment, rehabilitation, outcome prediction and monitoring during the adult stroke recovery journey. In particular, we were interested in AI in upper limb rehabilitation across all settings, from the acute to chronic phases of stroke management, including acute stroke units, rehabilitation facilities and community settings, as well as experimental laboratories.

Using our interactive dashboard (https://bit.ly/3X4k5HR), a researcher or stroke clinician can investigate the available data on a topic of interest. For example, if a person is interested in how AI is being used in stroke survivors with aphasia or speech problems, they can go to the topic cluster of “aphasia speech” and explore how AI is being used in relation to impairment, prediction or rehabilitation. Selecting that topic cluster, they would find 23 articles from 1990 to 2023. They could then explore what categories of application were available, e.g., assessment (n = 13) or assisted rehabilitation (n = 5). The details of the articles would be readily accessible, with potential filtering in relation to year of publication, journal, etc. The researcher could then investigate these articles more fully.

### 4.1. Progression of AI in Adult Stroke Rehabilitation

The evolution of AI technology in stroke rehabilitation has been a dynamic journey marked by significant milestones and breakthroughs over the decades. The conceptualization of AI dates back to 1956, when the field was officially founded at the Dartmouth Conference [167]. In the late 1950s, the introduction of ANN and the development of perception models laid the foundation for ML and pattern recognition. However, ANNs fell out of favor during the 1900s and 2000s [167]. In contrast, we observed the first conceptualization of AI application in adult stroke rehabilitation in the early 1990s (during the post-AI boom period), more than two decades after the AI technology was invented [167]. Early AI applications in this field primarily utilized less complex machine learning methods to predict outcomes and guide rehabilitation strategies. AI computational research underwent significant advancement since the mid-2000s, driven by developments in computational power, algorithmic innovation, and the availability of large datasets. Our review of research trends revealed a significant increase in the literature evaluating AI applications in adult stroke rehabilitation and recovery, particularly after 2016. These advancements have been driven by the need to overcome limitations in traditional rehabilitation methods, such as the shortage of skilled therapists and the subjectivity in patient assessments.

Over the last decade, AI applications have significantly improved clinical practices in adult post-stroke rehabilitation by innovating various aspects of care. Technological advancements, including AI, continue to be applied in stroke management to improve early detection, clinical outcome prediction and quick delivery of treatment by maximizing system efficiency from acute care [89,129,143]. The emergence of new approaches, such as AI-driven BCI, which leverages ANNs to interpret complex neurological signals, has enabled more precise assessments of motor function and cognitive recovery.

Another significant application of AI in stroke rehabilitation that emerged with the advent of the Internet of Things (IoT) during the late 2000s is the development of home-based rehabilitation and monitoring systems that leverage wearable sensor technology, facilitating remote monitoring and smart assistance and allowing for decentralized care models. Such innovations, including computer vision systems, have supported activity recognition, movement classification and clinical status prediction, thereby enhancing the accessibility and quality of rehabilitation services.

Additionally, AI has transformed imaging analysis, facilitating more precise evaluations of stroke lesions. Deep learning techniques in stroke imaging have been shown to enhance the accuracy of lesion detection and characterization, which is vital for predicting clinical outcomes. Such applications have also been linked to better prognostic assessments, helping to tailor rehabilitation protocols to individual needs. The timeline of AI technology, particularly the breakthroughs in deep learning algorithms, correlates with these innovations, as these methods provide the computational power and complexity needed to process clinical and sensor data. These advancements, combined with remote monitoring and intelligent digital assistants [160], pose opportunities for the wide adoption of telemedicine approaches in stroke care [168].

Furthermore, developments led automated post-stroke rehabilitation methods to become more sophisticated and efficient, facilitating the wide adaptation of systematic rehabilitation therapy through AI-driven virtual and robotic coaches, improving engagement among post-stroke survivors and motor recovery [96]. For example, the development of intelligent robotic systems designed for AI algorithms for upper limb and lower limb rehabilitation adapted to the specific needs of patients, providing personalized therapy that can improve motor function recovery [98,99,102,103]. Moreover, wearable systems integrated with AI enhanced motor rehabilitation by utilizing muscle electrical signals, thereby enabling targeted training of affected limbs [73].

AI applications in VR allowed for the construction of three-dimensional interactive immersive intelligent environments that facilitate rehabilitation exercises without the constraints of physical environments, thereby promoting a more engaging and effective recovery process [108]. These virtual interventions offer various advantages for stroke rehabilitation, such as enhancing neuroplasticity, providing intensive and engaging training, offering real-time feedback and simulating real-world activities. These advancements of AI applications in adult stroke rehabilitation and recovery have been marked by significant clinical advancements in precise, personalized rehabilitation approaches with broader outreach beyond traditional methods.

### 4.2. Upper Limb Rehabilitaion

Upper limb rehabilitation is a critical component of stroke recovery due to the significant impact that upper limb dysfunction has on daily living activities. A substantial proportion of stroke patients experience upper limb impairments at the onset of the stroke. We observed a significant number of studies (n = 310) across topics and themes related to upper limb rehabilitation. These AI applications in upper limb rehabilitation have shown their potential to enhance recovery outcomes and improve the precision, personalization and effectiveness of rehabilitation strategies. Below, we discuss ten notable studies on AI-assisted upper limb rehabilitation, as identified through our search.

One aspect of post-stroke upper limb rehabilitation is the timing and intensity of therapeutic exercises. In outpatient settings, the exercises may not be monitored or assessed by therapists, except during the follow-up assessments. This leads to the possibility that chronic stroke survivors are not engaging in the interventions as prescribed, resulting in suboptimal outcomes. To address such needs, Chae et al. [63] conducted a clinical trial with 22 stroke survivors employing a web-based upper limb telehealth system that utilizes a smartwatch and a digital health app. Motion data collected through the smartwatch’s IMU sensors were analyzed using CNNs that resulted in 99.9% accuracy in detecting the four selected exercise tasks based on bilateral movement therapy. By offering accessible and engaging rehabilitation activities, these systems improve patient compliance and outcomes in the long-term management of post-stroke upper limb impairments.

In remote monitoring and telerehabilitation, recognizing upper limb movements, assessing movement patterns and detecting deviations from normal motion or improvement in motor function are crucial for identifying specific impairments and their recovery, as well as tailoring interventions to address them. One notable approach proposed by Bijalwan et al. [118] used a hybrid deep learning approach to recognize physiotherapy exercises like extension, flexion and rotation, which are necessary for upper limb function recovery. By combining Kinect depth camera technology with ANN techniques like CNN and LSTM, the model showed significant pattern recognition accuracy of 98–100%. In a clinical study with 41 stroke survivors, Kim et al. [169] demonstrated the validity of utilizing a Kinect depth-sensing camera as a markerless approach to tele-access upper extremity function through FMA scoring. This system did not use any specialized devices other than the low-cost Kinect, and it provided a real-time virtual interface for therapists and patients. They used unsupervised learning and deep learning techniques like PCA and ANNs to predict FMA scores in a virtual home-based rehabilitation setup. Such approaches allow clinicians to track progress and adjust rehabilitation protocols in real-time without the need for frequent in-person visits.

AI applications with wearable sensor devices have shown promise in transforming upper limb rehabilitation. For example, Shull et al. [170] proposed a novel technique for hand gesture recognition and finger angle estimation using wrist-worn modified barometric pressure sensing. By leveraging this technology, an accurate assessment of hand movements and finger angles was predicted, offering a valuable contribution to upper extremity function evaluation and rehabilitation progress monitoring. Furthermore, Panwar et al. [117] introduced Rehab-Net, a deep learning framework that utilized wearable accelerometers to classify arm movements and provide real-time feedback during rehabilitation sessions. Their model achieved overall accuracies of 97.89% on semi-naturalistic data and 88.87% on naturalistic data, outperforming traditional handcrafted feature-based ML models. Patel et al. [124] introduced a novel approach that utilizes accelerometer data from wearable devices to monitor and assess the quality of upper limb movements in stroke survivors. Yang et al. [73] presented a sensor-enabled rehabilitation system that integrates a smart wearable armband, ML techniques, and a dexterous robot hand. This wearable technology offers a non-intrusive means of real-time tracking progress and providing personalized feedback and adaptive upper limb rehabilitation exercises tailored to individual needs, thereby optimizing the rehabilitation process.

The role of electromyography in this context cannot be overstated. EMG serves as a vital tool for assessing muscle activity and facilitating rehabilitation through various AI-driven applications. A notable approach involves using surface EMG signals in conjunction with ML techniques, as proposed by Cai et al. [74]. Their approach employed SVM for upper-limb motion pattern recognition using surface EMG signals to control a rehabilitation robot designed specifically for post-stroke patients. This technique showcases the potential of AI in automated robot-assisted rehabilitation exercises tailored to individual needs based on real-time muscle activity data.

AI applications in the real-time control of prosthetic devices using brain signals have the potential to recover adults’ upper limb motor function. Research indicates that BCIs can effectively promote neuroplasticity, which is crucial for motor recovery in stroke patients. Yanagisawa et al. [95] successfully decoded three hand movements from the EEG signals of a stroke survivor to control a prosthetic hand in real-time. This notable approach highlighted the possibility of integrating brain–machine interfaces to restore motor function and improve quality of life for individuals with severe upper limb impairments post-stroke.

Functional connectivity changes in the brain in the context of post-stroke upper limb rehabilitation have garnered attention due to their potential correlation with motor recovery levels after interventions. Várkuti et al. [171] highlighted the use of fMRI to identify connectivity patterns in the brain at rest, which may be indicative of neural cooperation and potentially linked to upper limb motor recovery. They predicted individual gains using functional connectivity changes based on the pre-post differences in fMRI measurements. This approach offers insights into the neural mechanisms underlying rehabilitation outcomes and could aid in tailoring personalized rehabilitation strategies for stroke survivors.

### 4.3. Lower Limb Rehabilitation

Our review also showed AI applications in lower limb impairment assessment, intervention and monitoring. Muscle weakness is a primary consequence of stroke, often leading to difficulties in performing activities of daily living, such as ambulatory activities. Studies have used human action recognition for ambulation monitoring [49,60] and virtual ambulatory task training [59]. In lower limb deficits, gait dysfunction is particularly notable, with patients frequently exhibiting altered gait patterns, such as hyperextension of the knee and reduced ankle dorsiflexion, which can increase the risk of falls and decrease overall mobility. Many studies have focused on gait and mobility, employing technologies such as EMG [76,77], marklerless computer vision [119], assistive exoskeleton robots [120], CT scanning [58] and wearable sensors [55,56,57]. For example, Cui et al. [51] developed an automatic gait analysis system to detect and quantitatively assess gait abnormality using ensembling classification, including SVM, random forest, KNN, naïve Bayes and ANN, which integrate motion captures, pressure sensors and EMG data. The clinical trial with stroke survivors and healthy participants showed 98.2% accuracy in classifying the severity of gait abnormality in a comparable score to the Wisconsin Gait Scale. Such automatic gait analysis systems can better facilitate clinical decision making, thereby enhancing the efficiency of rehabilitation treatments. Furthermore, specific gait alterations such as balance control [54], foot drop [52] and ankle–foot orthosis [53,135] can be detected, together with gait phases like swing [52]. These applications have enabled a holistic approach to gait and mobility rehabilitation, including the individualization of exercise programs.

### 4.4. Cognitive and Speech Rehabilitation

Cognitive deficits can manifest in various forms, including memory loss, executive dysfunction and attention deficits, which are prevalent in stroke survivors. Although a large proportion of research has focused on motor rehabilitation, we also observed AI applications in cognitive rehabilitation of adults, including assessment [45] and computer-assisted cognitive training [46,160]. One of the most promising applications of AI in cognitive rehabilitation is the use of VR [92,108]. Studies have shown that VR can create immersive environments that simulate real-life scenarios, allowing patients to practice cognitive skills in a controlled yet engaging setting.

The post-stroke speech rehabilitation research is primarily targeted towards aphasia, which can manifest as deficits in language and communication abilities. Innovative approaches such as AI-driven digital speech therapy are gaining traction [78,79,80,81], offering personalized treatment plans that cater to the diverse needs of patients with speech deficits, including dysarthria. These applications analyzed speech patterns and provided real-time feedback, enhancing the rehabilitation process. Moreover, recent advancements in AI have also led to integration with BCI, functional connectivity analysis and medical imaging techniques that facilitate and evaluate language abilities for individuals with speech impairments [81,147].

### 4.5. Limitations and Gaps in Current Methods

While AI has the potential to revolutionize adult stroke rehabilitation, some challenges and limitations still need to be addressed. These include the unavailability of standard datasets, diversity in assessment methods, high implementation costs, need for skilled resource personnel and equipment, technological limitations, safety and privacy of stroke survivors and ethical use of AI. For instance, automated rehabilitation systems may provide superior performance to conventional systems that rely on experienced therapists, but at a higher development cost. Studies have reported that VR or robotics-based systems can cost GBP 336–1602 per participant more than the conventional therapy, depending on the intensity [9]. Moreover, the successful implementation of real-world AI in stroke rehabilitation requires a multidisciplinary approach that involves technology and therapy skills. The absence of infrastructure can impede the implementation of AI-driven rehabilitation programs, limiting their reach and effectiveness. This includes insufficient computational resources and a lack of access to modern rehabilitation technologies such as immersive VR systems, sensors or robotic devices. For example, the use of markers for motion data collection will not be practical for automated supervision of at-home practices [172].

The safety and ergonomics of AI-driven assistive systems present another concern [172]. Of the two main forms of rehabilitation, i.e., inpatient rehabilitation in a hospital or rehabilitation facility and rehabilitation in the stroke survivor’s home, inpatient rehabilitation therapy, despite its drawbacks, poses very few risk factors regarding potential malfunctions or mishandling of equipment since the patients are constantly monitored by therapists [9,155]. For example, patients have given up using sensor devices during rehabilitation in the home as they were unfamiliar with technology systems and experienced difficulties in using them [63]. Moreover, the development of remote automatic stroke rehabilitation systems have relied on data collected from various sensors, including Microsoft Kinect, BTs Nirvana and Vicon optional tracker, which are not commonly available in outpatient environments [18]. Such factors remain a challenge in at-home rehabilitation, where the patient is minimally supervised by therapists.

Most rehabilitation systems rely on retrospective data and data collected from limited samples, typically ranging from a hundred to a few hundred, which may not adequately reflect the impairment profile of the stroke survivor population being targeted; potentially leading to biases in AI models [9,18]. In AI modeling, the quality and integrity of data is paramount. For example, we have used a prospective, well-phenotyped, longitudinal stroke cohort known as START to investigate clusters of impairment for survivors of stroke who are classified as mild according to the National Institute of Health Stroke scale [14]. Although the sample size was small (n = 73), with data mapped over three timepoints, meaningful interpretations were made using growing self-organizing maps [14].

Despite the many benefits of deep learning technology, another significant AI technology limitation is the ‘black box’ problem. Model interpretability varies significantly across the different types of AI algorithms. Deep learning methods are widely considered as the least interpretable AI algorithms. For example, in ANNs, human users find computational processes in the hidden artificial neural layers, which are difficult to interpret. More recent studies have investigated explainable AI techniques that help to understand the ‘black box’ problem [15,137]. For example, Zihni et al. [15] evaluated clinical feature importances and calculated Shapley values to interpret ML predictive models.

Finally, given the rapid acceleration of the field, it is recognized that all emerging AI technologies and applications may not be identified within the review period.

### 4.6. Review Methodology and Its Impact on Findings

In this review, we constrained the final dataset to AI applications in the context of stroke rehabilitation and recovery of adults. The process was iterative and involved review and consensus across AI and clinical experts from the authorship team at each step. The developed domain vocabulary and comprehensive search strategy conducted in clinical (e.g., MEDLINE, CINAHL) and computational (e.g., IEEE Xplore, ACM Digital Library) databases ensured that peer-reviewed articles published in English that combined methodological rigor with clinical relevance were included in the evidence synthesis. The exclusion of non-human studies, non-English language publications, non-adult focused studies and non-peer-reviewed articles further ensured the relevance within the scope of the review. It is beyond the scope of our review to detail the specific AI applications in upper limb rehabilitation, but practical reviews can be found elsewhere [155,173].

While this review is about adult stroke rehabilitation, it is important to acknowledge that there is a growing body of literature related to the pediatric population in similar areas. For example, specific robotic exoskeletons have been developed for the pediatric population [174].

### 4.7. Future Clinical Development Areas and Roadmap

Conventional stroke rehabilitation is a complex process involving multiple disciplines seamlessly transferring care from one to another or working together as a team. The current state of AI applications in the context of stroke rehabilitation is still in its infancy. To become a mainstream rehabilitation process, AI-based rehabilitation techniques need to be fully embedded in practice and should adopt the same principles of an integrated approach to rehabilitation rather than targeting one or other specific areas of impairment. The application of AI holds the potential to improve care delivery across boundaries in every aspect of stroke rehabilitation, offering stroke survivors enhanced chances for recovery and improved quality of life and possibly reducing the overall cost of care in future. However, realizing this potential requires further collaborative efforts from clinicians and technologists to address technical, ethical and practical challenges.

Ethical considerations play a crucial role in the deployment of AI in stroke rehabilitation [18]. Issues related to patient privacy, data security and the potential for algorithmic bias raise significant concerns. Furthermore, it may be necessary to establish limitations on the utilization of patient data, to regulate the origins of such data, and to ensure the validity, consistency and generalizability of the data needed to facilitate broader acceptance and implementation of AI applications in stroke rehabilitation.

There is a need for larger amounts of data and prospective clinical studies to build on successful AI-driven systems. However, the collection of large datasets also leads to concerns on the safety and privacy of patient data. The availability of large amounts of quality, clinically confirmed data is mandatory for further investigation of deep learning techniques.

In the context of telerehabilitation, an integrated strategy to gather real-world data should be actively sought. For example, many rehabilitation exercises could be enabled with AI-driven systems, precise diagnosis and training modalities. Rehabilitation protocols vary significantly based on functions targeted and the varying levels of impairment in post-stroke adults. The reliance on AI for clinical decision making may reduce human oversight, which is essential for addressing the nuanced needs of stroke patients. To achieve AI-driven personalized rehabilitation strategies, it is critical that researchers across multiple disciplines engage in collaborative efforts to streamline and tailor assessments and rehabilitation interventions.

Finally, there is a question about the level of acceptable accuracy of an AI system in healthcare [19]. Mistakes from automated systems can be prohibitive in gaining clinical acceptance. Therefore, further investigation is needed into the level of evidence and supporting data that will be sufficient to be relied on when implemented in rehabilitation facilities.

## 5. Conclusions

The complexity of stroke sequelae and the mystery of the human brain make rehabilitation challenging. With growing interest among clinicians, leveraging AI’s potential is promising for optimizing the recovery journey of stroke survivors. The impact of AI in adult stroke rehabilitation is evidenced across different areas, including improvements in physical function, cognitive abilities, speech, emotional well-being and overall quality of life. We believe that considered use of AI technologies may offer a transformative approach towards enhanced recovery trajectories and improved long-term outcomes for stroke survivors. Personalized interventions, leveraging neuroplasticity, automated therapy, including the use of sensor-based systems and robots, predicting outcomes, and telerehabilitation are key aspects that can be optimized through AI applications to improve adult stroke rehabilitation and recovery. Nevertheless, despite the potential benefits of AI technologies in enhancing rehabilitation outcomes, various challenges persist, including costs, skilled resources, technology and implications of its use. Collaborative effort is needed to realize the true benefit and role of AI in stroke rehabilitation and recovery.

## Figures and Tables

**Table 1:** Search terms used for conducting a comprehensive search across academic databases.

Base Search Terms (MeSH)
(“stroke”[MeSH Terms]) AND ((“artificial intelligence”[MeSH Terms]) OR (“machine learning”[MeSH Terms])) AND ((“stroke rehabilitation”[MeSH Terms]) OR (“neurological rehabilitation”[MeSH Terms]) OR (“neurosciences”[MeSH Terms]) OR (“Recovery of Function”[MeSH Terms])) AND (“adult”[MeSH Terms])
**Expanded Search Terms**
(“Artificial Intelligence” OR “Machine learning” OR “Deep Learning” OR “Computational Intelligence” OR “Machine Intelligence” OR “AI application*” OR “supervised learning” OR “unsupervised learning” OR “Artificial Neural Network*” OR “Computer Reasoning” OR “Computer Vision*” OR “Expert System*” OR “Natural Language Processing” OR “NLP”) AND (“Cerebral Vascular Accident” OR “Cerebrovascular Accident” OR “CVA” OR “Stroke” OR “Cerebrovascular Disorders” OR “Ischemic” OR “Brain attack” OR “Infarction” OR “Hemorrhagic” OR “Brain injury”) AND (“Rehabilitation” OR “Stroke rehabilitation” OR “neurological rehabilitation” OR “neurorehabilitation” OR “neuroscience” OR “Therapy” OR “Stroke Recovery” OR “Recovery” OR “Post Stroke” OR “Post-Stroke” OR “Poststroke” OR “profile” OR “profiling stroke” OR “trajectory recovery”) AND (“Adult*”)

**Table 2:** Article counts of research topics categorized by major research themes.

Research Themes and Topics	n	%
**Theme 1: Impairment**		
Functional impairment/capacity	115	16
Gait and mobility	83	12
Electromyography (EMG)/Motor impairment	44	6
Upper limb function	28	4
Speech	23	3
**Theme 2: Assisted Intervention**		
Brain–Computer Interface (BCI)	100	14
Rehabilitation/Robot-assisted therapy	61	9
Virtual reality (VR)	33	5
Computer vision	23	3
Deep learning-based systems	21	3
**Theme 3: Prediction**		
Outcome prediction	50	7
Data analysis for monitoring and service prediction	39	6
Motor function recovery	14	2
**Theme 4: Imaging and Neuroscience**		
Functional connectivity	40	6
Medical imaging	30	4

## Data Availability

The data that support the findings of this review are available via an interactive dashboard at https://bit.ly/3X4k5HR.

## Supplementary Materials

The following supporting information can be downloaded at: https://www.mdpi.com/article/10.3390/s24206585/s1, Supporting Information on AI-assisted article screening. Table S1: Prompts sent to Open AI GPT 3.5-Turbo LLM model for the screening of title and abstract in rounds one and two.; Table S2: Confusion matrix for LLM-driven automatic screening of titles and abstracts for rounds one and two. The comparison is based on a sample of the dataset (n = 200). References [175,176] are cited in the Supplementary Materials.

## Appendix A

Table A1 shows the clinical vocabulary developed with domain experts and then expanded using the collected data by applying NLP techniques.

**Table A1:** The developed clinical vocabulary for artificial intelligence, stroke, rehabilitation and recovery.

Artificial Intelligence	Stroke	Rehabilitation	Recovery
Artificial intelligence (AI) Machine learning (ML) Deep learning Computational Intelligence Machine intelligence AI application Supervised learning Unsupervised learning Classification Artificial neural network (ANN) Computer reasoning Computer vision Computer intelligence Cognitive computing Expert systems Natural language processing (NLP) Robotic intelligence Smart machines Synthetic intelligence Intelligent systems Automated intelligence Reinforcement learning Fuzzy logic Decision trees Classification Clustering Regression Bayesian networks Genetic algorithms Sentiment analysis Speech recognition Image processing Data mining Predictive analysis Sentiment analysis	Stroke Cerebrovascular accident (CVA) Cerebral vascular accident Cerebrovascular disorders Ischemic Hemorrhagic Brain attack Brain injury Infarction Neuroscience Adult	Rehabilitation Neurological rehabilitation Neurorehabilitation Therapy Language Skill Movement Activity Sensation Learning Motor Cognition Training Function Task Participation Performance Rehab Telerehabilitation Cognitive Speech Sensory Somatosensory Intervention	Recovery Profile Trajectory Impairment Stroke recovery Post stroke Post-stroke Poststroke Human Relearning Independence Function Quality of life Emotional well-being Engagement Adaptation
