# Supplementary material for: AI Applications in Adult Stroke Recovery and Rehabilitation: A Scoping Review Using AI
Source: Sensors (Basel). 2024 Oct 12;24(20):6585. doi: 10.3390/s24206585 (PMC11511449; doi:10.3390/s24206585)
Supplement: Supplementary file 1 [file sensors-24-06585-s001.zip › sensors-3209007-supplementary.pdf]

Supplementary Materials S1: Artificial Intelligence-Assisted Article Screening

In the automated screening process, we adopted an interactive approach by employing Open AI ChatGPT 3.5-Turbo large language model (LLM) [175]. Lare language models can respond to free-text queries without being specifically trained in the task in question and have proven to be a valuable tool in various fields, including healthcare research [176]. To ensure effective screening, we carefully prepared queries (prompts) using the study's inclusion/exclusion criteria and programmatically prompted them against ChatGPT 3.5-turbo model to generate responses in a conversational manner (Table S1).

The process was conducted in two rounds: (1) Prompt development. Programmatically prompting GPT-3.5 Turbo LLM model with the entire keyword-filtered dataset and manual validation of a sample (n = 200) of results (by I.S., B.H.). Exclusion of records based on the automatic classification. (2) Development of fine-tuned prompt. Programmatically prompting GPT-3.5 Turbo LLM model with the filtered dataset from round one. Manual validation of results sample (n = 200) by I.S. and B.H.. Table S2 shows the confusion matrix for rounds one and two as an automatic classification performance measure. Results of automatic screening round two were used as a reference for assessing eligibility manually by B.H., K.J.L. and J.S.

Table S1. Prompts sent to Open AI GPT 3.5-Turbo LLM model for the screening of title and abstract in rounds one and two.

**Automated Screening Round 1 Prompt**
[{"role": "system", "content": "Classify if the given text mentions using artificial intelligence for stroke rehabilitation and recovery of adults. Answer yes/no only."},<br>{"role": "user", "content": <title and abstract> }]
**Automated Screening Round 2 Prompt**
[{"role": "system", "content": """You are a researcher rigorously screening titles and abstracts of scientific papers for inclusion or exclusion in a review paper. Use the criteria below to inform your decision. If any exclusion criteria are met, must exclude the article. Include the article only if all inclusion criteria are met, Only type ″included″ or ″excluded″ to indicate your decision. Do not type anything else.<br>Inclusion criteria: 1. Studies must directly focus on the rehabilitation and recovery of stroke survivors; encompassing neuroscience, neurorehabilitation, profiling, assessment of severity or impairment, therapy, monitoring, treatment, assistive robots, recovery, prediction, swallowing difficulties, dysarthria, gait training, robotic intervention. 2. Studies must focus on Artificial Intelligence-based applications for stroke rehabilitation and recovery, (including virtual reality, robotics).<br>3. Studies must focus on adult stroke survivors (humans) encompassing elderly.<br>Exclusion criteria: 1. Studies do not focus on the rehabilitation and recovery of stroke survivors. (e.g. stroke identification, diagnosis) 2. Studies that do not apply Artificial Intelligence.<br>3. Studies focused on non-adults encompassing newborns, Infants, children, Adolescents."""},<br>{"role": "user", "content": "Title:"+ <title> +"\n Abstract:"+ <abstract> }]

Table S2. Confusion matrix for LLM-driven automatic screening of titles and abstracts for rounds one and two. The comparison is based on a sample of the dataset (n = 200).

	**Manual Screening Result**	
**Round 1 Automatic Screening Result**	Include	Exclude
Include	37	63
Exclude	0	100

	Manual Screening Result	
**Round 2 Automatic Screening Result**	Include	Exclude
Include	30	3
Exclude	8	159

Supplementary Materials S2: Common Artificial Intelligence Techniques

1. Introduction

Artificial Intelligence (AI) encompasses a domain within computer science that is focused on the development of machines and systems capable of mimicking human intelligence. Through the application of methodologies such as machine learning (ML), artificial neural networks (ANNs), and natural language processing (NLP), AI facilitates the execution of tasks by computers that ordinarily necessitate human-level cognitive processes, including but not limited to problem-solving, decision-making, and language comprehension.

Machine learning is a subfield of AI. Machine learning algorithms can be classified into three distinct categories: supervised learning, unsupervised learning, and reinforcement learning (RL). Traditional ML algorithms need handcrafted input data attributes (features) to learn from. Therefore, traditional ML modelling experiments start with feature pre-processing and feature selection stage to extract the most useful attributes of the data which are then fed into the algorithm.

2. Supervised Learning

In supervised learning, the predicted outputs are known and used to train the models. Supervised ML modelling has two phases: training and testing. The labeled training data helps predict outcomes via techniques such as classification, regression, predictive modelling and ensemble methods. Predicting impairment levels uses supervised learning since ML algorithms can be trained on already available labeled data to figure out patterns. In the testing phase, the label of new data can be predicted based on the learnt data patterns. This can also be considered as learning a parameterized data function (model) that can map input features to its target categorical variable (label). In the training phase, the ML algorithm learns the parameters of this function through the data provided. The most common supervised learning techniques are classification and regression.

2.1. Classification Algorithms

In classification, the ML algorithm is trained to allocate given data to a specific discrete label. Support vector machines (SVM), k-nearest neighbors (KNN), decision tree (DT), and random forest are some of the popular classification algorithms.

2.1.1 Support Vector Machines (SVM)

The SVM algorithm segregates a dataset by drawing a hyperplane or reference line for the purpose of data categorization. SVM determines the appropriate side of the delineated boundary to which new data is assigned, based on the earlier categorized data. The hyperplane or line is positioned as distantly from each individual data point as possible. SVM offers the benefit of providing a visual representation of the outcomes of data classification. While SVM serves as robust classifiers, they have some drawbacks. For example, the inner mechanisms underlying SVM may pose comprehension challenges due to complex mathematical constructs; therefore, interpreting the model can be challenging [17].

2.1.2 K-Nearest Neighbors (KNN)

The KNN algorithm represents a straightforward yet highly efficacious classification methodology predicated upon the intuitive notion that analogous data points are situated in close spatial proximity according to a given metric. In particular, KNN evaluates the degree of similarity among data points by employing the Euclidean distance among the K nearest data points. The most favorable value of K may be determined experimentally across a range of values utilizing the classification error. k-nearest neighbors is extensively used in scenarios where a search query is conducted, necessitating that the resultant outputs exhibit similarity to an existing entity. K-nearest neighbors is popular since it is free from assumptions about the underlying data distribution [155].

2.1.3 Decision Tree (DT)

A decision tree is trained by identifying optimal characteristics and a critical threshold that effectively discriminates the attributes of the input data into the target label categories. Decision tree serves the dual purpose of addressing both classification and regression problems. A DT is characterized as a collection of conditional if-then-else statements featuring multiple branches interconnected by decision nodes and concluded by terminal leaf nodes. The decision node signifies the juncture at which the tree splits into various branches, with each branch representing a specific decision made by the algorithm, while the leaf nodes denote the output of the model. This output may take the form of a categorical label in classification scenarios or a continuous numerical value in the context of regression. An extensive array of decision nodes is used in this manner to construct the DT. The primary goal of DT is to effectively capture the input-output patterns while utilizing the most concise tree structure possible.

2.1.4 Random Forest

Random forest is an ensemble algorithm that includes an array of individually trained DTs [17]. Each component DT predicts a classification output. The random forest algorithm employs a stochastic process to select an array of DTs and subsequently identify a model that consists of a collection of DTs featuring the most favourable features. The final classification outcome is decided from the maximum number of votes based on DTs' classification decisions. A notable advantage of the random forest algorithm lies in its capability to ascertain the classification of each individual input data instance; however, it presents the drawback of being inherently challenging to visualize.

2.2. Regression Algorithms

In regression, algorithms can predict a continuous value for a given input data set. In fact, both linear and logistic regressions can be considered regression algorithms.

2.2.1 Logistic Regression

Logistic regression is an algorithm that employs the principles of linear regression methodology for the purpose of classification [17]. This methodology delineates the linear associations that exist between a minimum of one independent variable and a singular dependent variable. The dependent variable is ascertained through the computation and application of weights attributed to each independent variable. A logistic regression model offers both classification capabilities and probability estimations, thereby enhancing the understanding of each variable's contribution to the model fit.

3. Unsupervised Learning

In unsupervised learning, the desired output is unknown, and the objective is to discover structure in the data, not to generalize a mapping from inputs to outputs. In other words, unsupervised learning algorithms extract data patterns or relationships without any guidance as to the "correct" answer. This technique is used to discover hidden patterns in data without human intervention and commonly relies on dimensionality reduction and clustering.

3.1. Dimensionality Reduction

Dimensionality reduction algorithms aim to simplify datasets while preserving their essential characteristics. This technique transforms high-dimensional data into a lower-dimensional space, facilitating easier visualization, interpretation, and analysis. Popular algorithms include principal component analysis (PCA) and independent component analysis (ICA).

3.1.1 Principal Component Analysis (PCA)

Principal component analysis is a widely utilized statistical technique in data analysis, particularly for dimensionality reduction. It aims to transform a dataset into a new coordinate system, where the greatest variance by any projection of the data lies on the first coordinate (the first principal component), the second greatest variance on the second coordinate, and so forth. This transformation is achieved through the computation of eigenvectors and eigenvalues from the covariance matrix of the data, which allows for the identification of the directions (principal components) that maximize the variance in the dataset.

3.2. Clustering

In clustering, the algorithm is presented with unlabeled data, subsequently it learns to group similar data points together. Following this training phase, validation methodologies are employed to optimize the model's parameters. Commonly used clustering algorithms include k-means and self-organizing maps (SOM).

3.2.1 K-means

The k-means algorithm iteratively refines the cluster assignments into a predetermined number of clusters (denoted as ‘K’ number of clusters) until convergence is achieved. This iterative process begins with the random initialization of cluster centroids, followed by assigning data points to the nearest centroid and subsequently updating the centroids based on the mean of the assigned points. The k-means algorithm is particularly valued for its simplicity and efficiency, making it applicable across various scenarios.

3.2.2 Self-Organizing Maps (SOM)

Self-organizing maps is a class of artificial neural networks that utilize unsupervised learning to map high-dimensional input data into a lower-dimensional representation, typically a two-dimensional grid, while preserving the topological properties of the data. This characteristic allows similar input data to be represented by nearby nodes on the map, facilitating visualization and clustering of complex datasets. A notable related algorithm is growing self-organizing maps (GSOM), which addresses some of SOM's limitations.

4. Reinforcement Learning

Reinforcement learning (RL) algorithms are trained through trial-and-error procedures in order to take action. The fundamental principle of RL is based on the idea of learning through trial and error, where agents receive feedback in the form of rewards or punishments as they perform actions in various states of the environment. This feedback mechanism is crucial for the agent to adjust its behavior to maximize cumulative rewards over time. Unlike supervised learning, RL does not necessarily use a training dataset.

The need for RL algorithms often arises in the real world, and they require efficient learning and careful definition of input values and exploration strategies. RL is a common application in AI-driven robotic devices.

5. Deep Learning

Deep learning consists of artificial neural networks, a mathematical and computational model that is inspired by the structure and functional aspects of biological neural systems. Artificial neural networks consist of interconnected nodes (artificial neurons) processing information using a connectionist computational approach. This network adaptively changes its structure based on external or internal information, which flows during the learning phase, forming a robust dynamic model of the complex

relationships between inputs and outputs or patterns in data. Unlike traditional ML methods that require handcrafted predictive features as input, deep learning methods learn these features adaptively from the data.

Artificial neural networks have been widely applied in complex data analysis tasks including computer vision, NLP, speech recognition, sensor signal processing and medical imaging data. Common algorithms include convolutional neural network (CNN), recurrent neural network (RNN), Long Short-Term Memory (LSTM) and graph neural networks (GNN).

Convolutional neural networks are designed to automatically and adaptively learn spatial hierarchies of features from images, making them particularly effective for tasks such as image classification and object detection in computer vision.

6. Neuro-Fuzzy Models

Fuzzy logic is a significant branch of AI that deals with reasoning that is approximate rather than fixed and exact. Fuzzy logic algorithms extend classical logic by allowing for degrees of truth rather than the usual binary true/false values. It provides a framework for incorporating human-like reasoning into computational models, allowing for more flexible and interpretable decision-making processes. This characteristic makes fuzzy logic particularly useful in situations where information is uncertain or imprecise.

7. Computer Vision

Computer vision enables machines to interpret and understand visual information from the environment, primarily applying AI techniques with images and videos. It encompasses a variety of tasks such as image classification, object detection, and semantic segmentation, which are essential for applications ranging from medical imaging to markerless motion analysis and telerehabilitation systems. Deep learning techniques, particularly CNNs has emerged as a dominant technique in computer vision.

8. Natural Language Processing (NLP)

Natural Language Processing is a subset of AI that focuses on the interaction between computers and human language. It encompasses a range of computational techniques aimed at enabling machines to understand, interpret, and generate human language meaningfully. NLP techniques have been commonly employed in aphasia speech rehabilitation research.
